# Analysis and validation of macromolecular *B* values

**DOI:** 10.1107/S2059798319004807

**Published:** 2019-04-30

**Authors:** Rafiga C. Masmaliyeva, Garib N. Murshudov

**Affiliations:** a Institute of Molecular Biology and Biotechnology ANAS, Matbuat Avenue 2a, Baku 1073, Azerbaijan; b MRC Laboratory of Molecular Biology, Francis Crick Avenue, Cambridge CB2 0QH, England

**Keywords:** macromolecular structures, *B* values, inverse-gamma distribution, maximum-likelihood estimation

## Abstract

This paper describes a global analysis of macromolecular *B* values. It is shown that the distribution of *B* values generally follows the shifted inverse-gamma distribution (SIGD). The parameters of the SIGD are estimated using the Fisher scoring technique with the expected Fisher information matrix. It is demonstrated that a contour plot based on the parameters of the SIGD can play a role in the validation of macromolecular structures. The dependence of the peak-height distribution on resolution and atomic *B* values is also analysed. It is demonstrated that the *B*-value distribution can have a dramatically different effect on peak heights at different resolutions. Consequently, a comparative analysis of the *B* values of neighbouring atoms must account for resolution. A combination of the SIGD, peak-height distribution and outlier detection was used to identify a number of entries from the PDB that require attention. It is also shown that the presence of a multimodal *B*-value distribution often indicates that some loops or parts of the molecule have either been mismodelled or have dramatically different mobility, depending on their environment within the crystal. These distributions can also indicate the level of sharpening/blurring used before atomic structure refinement. It is recommended that procedures such as sharpening/blurring should be avoided during refinement, although they can play important roles in map visualization and model building.

## Abstract 2

This paper describes some statistical tools for analyses of macromolecular *B* values.

## 1. Introduction   

Refinement and validation of atomic models elucidated using crystallographic, and more and more increasingly single-particle cryo-EM, methods (Frank, 2006 ▸) are essential steps in the derivation of reliable three-dimensional structures of macromolecules. Atomic refinement procedures based on Bayesian statistics are now routine (Bricogne, 1997 ▸; Murshudov *et al.*, 2011 ▸; Pannu & Read, 1996 ▸). Prior structural and chemical information pertaining to building blocks of macromolecules are used during refinement (Vagin *et al.*, 2004 ▸; Long *et al.*, 2017 ▸; Moriarty *et al.*, 2009 ▸; Nicholls *et al.*, 2012 ▸; Smart *et al.*, 2011 ▸, 2012 ▸) as well as for validation (Davis *et al.*, 2007 ▸; Read *et al.*, 2011 ▸). This aids the derivation of chemically and structurally sensible atomic models that are consistent with prior knowledge, whilst transferring as much information from the experimental data to the model as possible via the likelihood function. There are a number of research papers and software tools that are dedicated to the validation of positional parameters of atomic models (see Chen *et al.*, 2010 ▸; Hooft *et al.*, 1996 ▸; Read *et al.*, 2011 ▸, and references therein). These papers and the corresponding software tools have been instrumental in improving the quality of the models deposited in the PDB (Read *et al.*, 2011 ▸; Berman *et al.*, 2002 ▸). As highlighted by Pozharski *et al.* (2013 ▸) and Weichenberger *et al.* (2013 ▸), there is still a long way to go before we can claim that the quality of the models in the PDB agrees well with prior knowledge and optimally reflects experimental data.

Parameters of atomic models include positions, occupancies and isotropic/anisotropic atomic displacement parameters (ADPs; also known as *B* values, *U* values and temperature factors). While many research papers have been dedicated to the validation of ADPs (see Read *et al.*, 2011 ▸, and references therein), works dedicated to the analysis of ADPs are few and far between (Dauter *et al.*, 2006 ▸; Merritt, 2011 ▸, 2012 ▸; Negroni, 2012 ▸;). Moreover, ADPs are often used as a pr­oxy for model quality, for example for the selection of reliable portions of macromolecules, and for validation (Chen *et al.*, 2010 ▸). There are at least two problems with such procedures.

(i) ADPs contain information about crystal disorder, the relative mobility of atoms within a molecule, as well as absorbing many shortcomings of the modelling and parameterization (*e.g.* errors in atomic positions).
(ii) The absolute values of ADPs are not meaningful. For example, by sharpening or blurring the data before refinement one can decrease or increase the average ADPs arbitrarily. Rather, it is more appropriate to analyse the relative values of the ADPs within the molecule and/or crystal/cryo-EM structure.

The resolution at which the structure has been resolved should also play a role in the analysis of *B* values. For example, a *B*-value difference of 10 Å^2^ would have a completely different effect on the shape and height of the density were the data to extend to a resolution of 3 Å compared with 2 Å. At 3 Å resolution this 10 Å^2^ difference would have little effect on the shape and height of the density, whereas at higher resolutions a difference of 10 Å^2^ would have a more dramatic effect. The relative difference between *B* values is also of importance; the effect of atomic *B*-value differences of 10 versus 20 Å^2^ will be more dramatic than 100 versus 110 Å^2^. Moreover, the distribution of *B* values should be considered in the analysis of *B* values and the selection of reliable portions of the structure. One should always remember that over-sharpening can make some ADPs negative, making them physically unreasonable. On the other hand, too much blurring may result in loss of structural details in the density maps.

In this contribution, we will describe some statistical tools for the analysis of isotropic *B* values. Firstly, we explore using the shifted inverse-gamma distribution (SIGD) to describe the distribution of ADPs within one crystal structure, as well as for each chain within the molecule. We then analyse the distribution of atomic peak heights for point atoms at different *B* values and resolutions, and then describe the application of these techniques to selected entries from the Protein Data Bank (PDB; Berman *et al.*, 2002 ▸).

It should be noted that the inverse-gamma distribution has previously been suggested (Dauter *et al.*, 2006 ▸) and used (Negroni, 2012 ▸) for the analysis of macromolecular structures. Here, we extend these analyses and add new statistics: an atomic peak-height distribution for point atoms that is dependent on resolution and *B* value, which reflects the effect of *B* values on the density better than mere *B* values. We also present a new plot based on SIGD parameters that helps in the visual identification of suspect crystal structures.

## 2. Shifted inverse gamma as a model for the *B*-value distribution   

Individual atomic ADPs are proportional to the variances of positional parameters; ADPs represent the mobility of atoms as well as the accuracy of the positional parameters. In Bayesian statistics, the inverse-gamma distribution (IGD) is often used as a prior probability distribution to model the variance of a normal distribution (see, for example, O’Hagan, 1994 ▸). Since ADPs are proportional to the variances, we can postulate that it is likely that the distribution of ADPs will resemble the IGD. However, since average *B* values depend on the sharpening level of the data, we add an additional shift parameter to the IGD. Sharpening/blurring should change the average *B* value without affecting the shape of the distribution, except in cases where over-sharpening produces negative or many small *B* values. Therefore, we assume that the distribution of isotropic ADPs can be modelled using the SIGD, 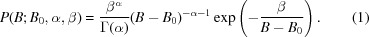
This distribution has three parameters: shape (α), scale (β) and shift (*B*_0_). If there is no over/under-sharpening of Fourier coefficients then *B*_0_ = 0, although this is rarely the case. Changing ADPs from *B* to *u* = *B*/8π^2^ only affects the scale and shift parameters. Since the shape parameter is also known as the degrees of freedom, it is tempting to assume that since the positional parameters of atoms reside in a three-dimensional space, the shape parameter of the SIGD would be around 3. However, we refine this parameter using soft harmonic restraints to ensure that the estimation of SIGD parameters is stable while allowing some variability.

**Figure 1:**
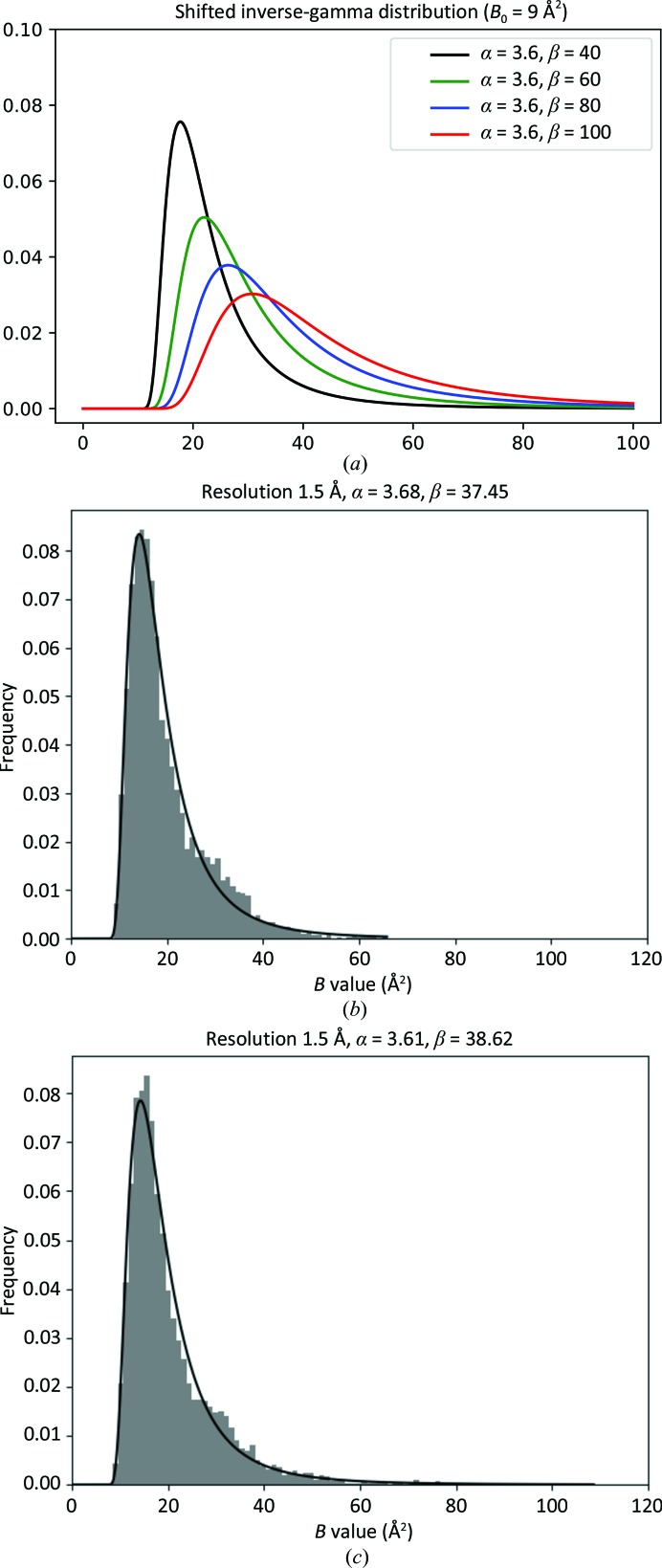
The shifted inverse-gamma distribution (SIGD). (*a*) SIGD with various parameters. (*b*) An example of the *B*-value distribution of a model from the PDB (PDB entry 1a4i) with the corresponding SIGD distribution; restrained refinement was performed. (*c*) The same example as in (*b*) but with unrestrained refinement.

Fig. 1 ▸ shows the SIGD for different shape and scale parameters (Fig. 1 ▸ *a*) and the empirical frequency distribution of *B* values for an example from the PDB (PDB entry 1a4i; Figs. 1 ▸ *b* and 1 ▸ *c*, corresponding to restrained and unrestrained refinement, respectively). This figure demonstrates that, at least for this particular case, the empirical *B*-value distribution resembles the SIGD reasonably well. Comparison of Figs. 1 ▸(*b*) and 1 ▸(*c*) exemplifies how unrestrained refinement does not change the distribution of *B* values dramatically, at least at this resolution, in this case.

### 2.1. Maximum-likelihood refinement of inverse-gamma parameters with harmonic restraints   

For each PDB entry in the test set, we refined the SIGD parameters by maximum-likelihood estimation using the Fisher scoring method with the expected Fisher information matrix (Stuart *et al.*, 1999 ▸; Steiner *et al.*, 2003 ▸). The derivation of the maximum-likelihood equations for this case is given in Appendix *A* [App appa]. Starting values for the parameters are derived using the relationship between the parameters of the SIGD and its mean and variance. Parameter estimation using the first few moments of the distribution is known as the method of moments (Stuart *et al.*, 1999 ▸), 
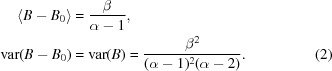
Note that the mean of the SIGD exists only if α > 1, and the variance exists only when α > 2. We have two equations and three parameters to estimate. We choose the starting value of *B*_0_ to be equal to 90% of the minimum of the ADPs in the PDB file considered, thus reducing the number of unknown variables to two. Equations (2) are solved to find the initial values as
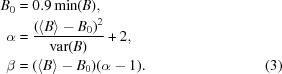
These starting values are iteratively improved using Fisher’s scoring method (Appendix *A* [App appa]) until convergence is reached. During the estimation process, care was taken to make sure that the estimates are reasonable, *i.e.* shape parameters are restrained to be near 3.5 and shift parameters are constrained to obey *B*_0_ < 0.9*B*_min_. Note that a negative value of *B*_0_ is an indication that the observed data may have been over-sharpened beyond reason, in which case parameter estimation becomes unstable.

## 3. Resolution- and *B*-value-dependent peak height at the centre of atoms   

Since the 3D maps used for model building in crystallographic and cryo-EM experiments correspond to densities of electrons and electrostatic potentials, respectively, it is interesting to analyse the effect of *B* values on the peak height at the centre of atoms for a given resolution. It is clear that the peak heights are dependent on atom types, occupancies, resolution and *B* values. In order to allow comparison of atomic peak heights, we ignore the effect of different atom types and occupancies; we essentially treat all atoms as point atoms^1^. As a result of resolution cutoff and *B* values (atomic mobility), the density becomes smeared out; this affects the values of the density map at the centre of atoms. The density corresponding to the point atom with *B* value equal to *B*_mod_ and resolution *s*_max_ = 1/*d*_max_ can be calculated using (see, for example, Chapman, 1995 ▸)
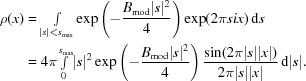
This is the shape of the point-atom density corresponding to a given resolution and *B* value. The density at the centre can be calculated by letting *x* = 0:
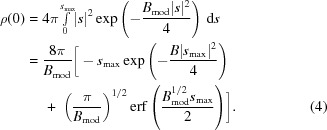
As can be seen, the density at the centre of the atoms depends on the resolution as well as on the *B* value. The real observed density will also depend on the noise level, the weights used in map calculations, the occupancies of the atoms, the quality of the amplitudes and phases, the number, types and proximity of neighbouring atoms, the overall anisotropy of the data and many other factors. However, a very simple analysis of peak heights should shed some light onto what can be expected at a given resolution. Even if the distribution of the *B* values is known, it is tricky to derive a closed-form expression for the distribution of peak heights at the atomic centre; therefore, in the following analysis we will use only empirical and simulated distribution histograms for peak-height analysis.

**Figure 2:**
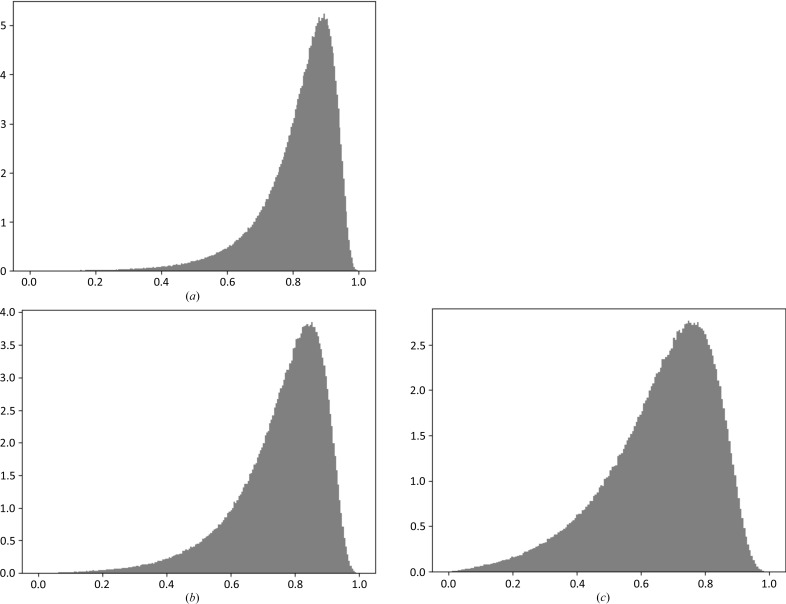
Resolution- and *B*-value-dependent peak-height distribution. The SIGD, with parameters α = 3.6, β = 32, corresponds to the model with PDB code 1fce. This model was used to generate peak-height distributions at (*a*) 3 Å, (*b*) 2.5 Å and (*c*) 2 Å.

Fig. 2 ▸ shows the histogram of *B* values for one example and the corresponding peak-height distribution calculated at different resolutions. It is seen that although the *B*-value distributions are the same, changing the resolution dramatically changes the distribution of peak heights. At higher resolution, as might be expected, small differences in *B* values have a much more dramatic effect on the density level than at lower resolutions. Therefore, it is always a good idea to consider the *B*-value distribution together with resolution, or to consider how the *B*-value distribution and its effect on peak heights depend on resolution.

## 4. Selection of the PDB entries   

We considered the 89 862 entries from the PDB, as of December 2016, for which the experimental method was X-ray crystallography. For further analysis, we used only the models for which the high-resolution diffraction limit is between 1.5 and 3 Å. To avoid dealing with noncrystallographic symmetry constraints, the use of which is not always clear from the PDB entry, we removed virus structures. Of the remaining models, we were able to refine 46 952 automatically using the refinement program *REFMAC*5 (Kovalevskiy *et al.*, 2018 ▸) available from *CCP*4 (Winn *et al.*, 2011 ▸). Reasons for refinement failure include (i) the ligand present in the PDB was not in the CCP4 monomer library at the time of refinement (this was the most common case), (ii) no structure-factor amplitudes^2^ and (iii) space-group inconsistencies between the PDB file and the reflection-data file. The remaining crystal structures contained roughly 160 000 chains, among which there were 145 800 protein chains. We also excluded cases with *R* factors higher than 35%. We used these crystal structures and corresponding chains for further analysis. Table 1 ▸ gives a short summary of the selection of PDB entries.

Table 2 ▸ lists the entries used as examples in this work. It should be stressed that the aim of this contribution is not to criticize a particular PDB entry; rather, we would like to highlight the shortcomings of the techniques used at the time of elucidation of these macromolecular structures.

## 5. Results and discussions   

### 5.1. Overall parameters of the shifted inverse-gamma distribution   

**Figure 3:**
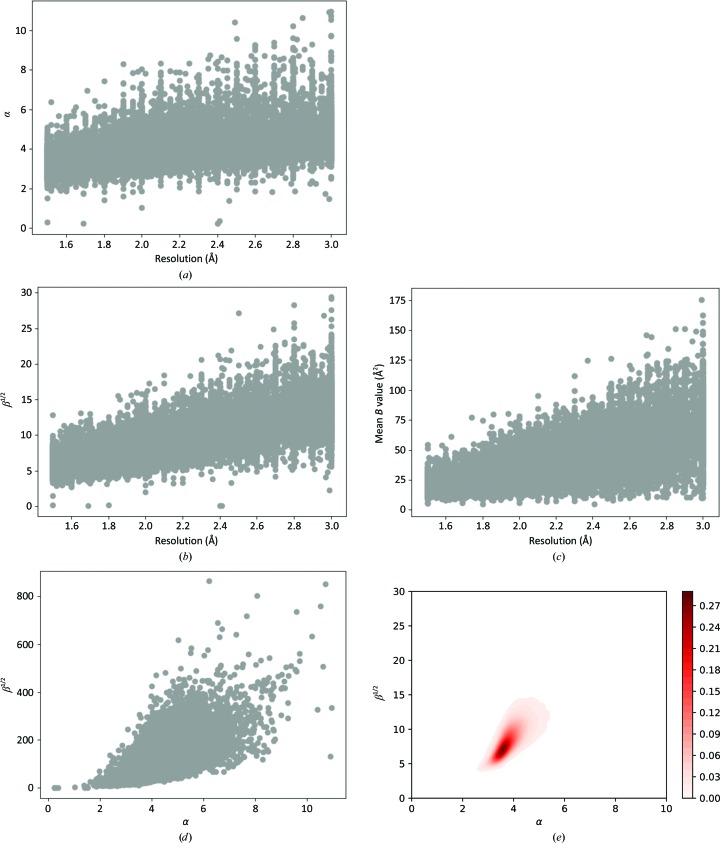
The dependence of SIGD parameters on each other and on resolution. (*a*) Dependence of α on resolution. (*b*) Dependence of β^1/2^ on resolution. (*c*) Dependence of average *B* value on resolution. (*d*) Scatter plot of α and β^1/2^, referred to as the α/β plot. (*e*) Smoothened distribution of α and β^1/2^.

We applied the method described above for the estimation of SIGD parameters for all selected PDB entries. The method was implemented in the statistical package *R* (R Core Team, 2018 ▸) and was later ported to Python. Figs. 3 ▸(*a*) and 3 ▸(*b*) show the shape and scale parameters versus resolution. For completeness, we also show average *B* value versus resolution (Fig. 3 ▸ *c*). It is evident that both α and β depend on resolution.

The plot of α versus β^1/2^ (Figs. 3 ▸ *d* and 3 ▸ *e*) shows the co-dependency of these two parameters. Moreover, very high values of α or β are an indication that either suboptimal refinement was performed or the data have been massaged (whether intentionally or not) before refinement was performed. If α and β are outside the region defined in Fig. 3 ▸(*e*) then the model should be considered for re-refinement with careful consideration of atom types as well as potentially erroneous loops. We find this plot to provide useful information regarding refinement behaviour. In all our following analyses we use this plot to identify peculiar PDB entries.

### 5.2. Over-sharpened cases   

**Figure 4:**
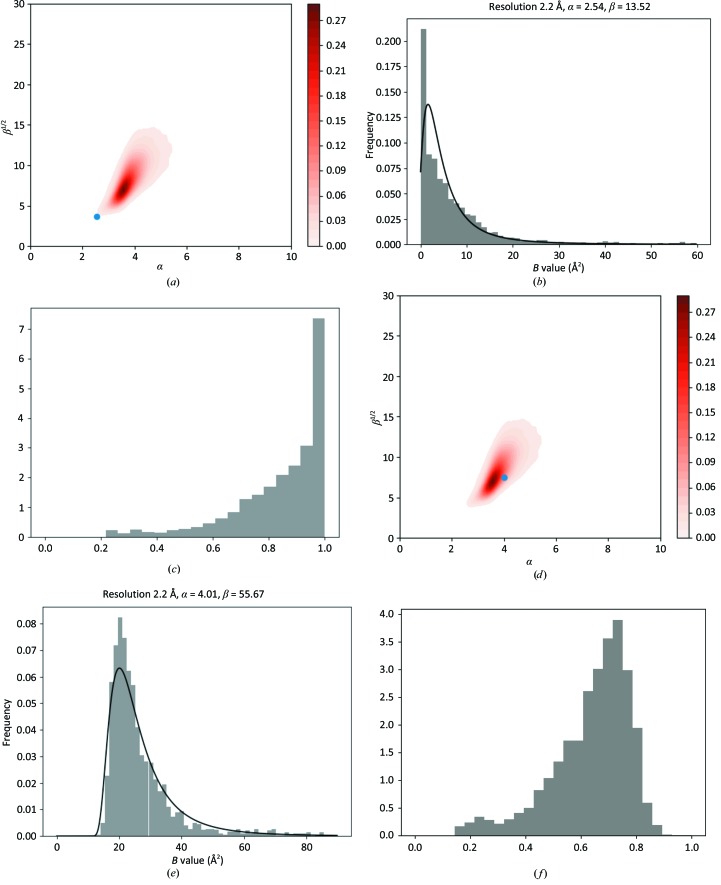
An over-sharpened case: PDB entry 3ad4. (*a*) Parameters of the SIGD for PDB entry 3ad4 shown on an α/β plot. (*b*) The SIGD before blurring. (*c*) The peak-height distribution before blurring. (*d*) The SIGD parameters on an α/β plot after blurred refinement. (*e*) The SIGD after blurred refinement. (*f*) The peak-height distribution after blurred refinement.

When the *B*-value distribution is shifted towards the left with a floor at a certain *B* = *B*_min_ and a large number of atoms all have the same small *B* value (Fig. 4 ▸ *d*) then it is an indication that the data have been over-sharpened before refinement. In this case, the parameter α becomes small or refinement of the SIGD parameters becomes unstable. There are a few such cases in the PDB; here, we use PDB entry 3ad4 as an example. Fig. 4 ▸ shows the SIGD parameters on the α/β plot (Fig. 4 ▸ *a*) and the histogram with the associated SIGD distribution (Fig. 4 ▸ *b*), as well as the corresponding peak-height distribution (Fig. 4 ▸ *c*) for this case.

For the purposes of visualization and model building, slight over-sharpening can be an acceptable procedure if care is exercised with the interpretation of water molecules; the effects of series termination and noise amplification can result in peaks that could be incorrectly interpreted as waters. In such cases, a careful analysis of contacts must be performed in order to avoid modelling wrong ‘water’ molecules. However, for refinement, over-sharpening can result in suboptimal atomic models as some of the atoms may have negative *B* values. Almost all existing refinement programs use some lower threshold (for example 0.5–2 Å^2^) in order to avoid dealing with negative *B* values. As a result, the *B*-value distribution becomes distorted, making interpretation of the *B* values (and sometimes the whole molecule) difficult. When TLS refinement is also performed then the behaviour of *B* values can become even more unpredictable, resulting in large negative or positive peaks reflecting wrong atomic *B* values.

For our example case, we added a small *B* value (20 Å^2^) and re-refined the model, resulting in the *B*-value distributions shown in Figs. 4 ▸(*d*), 4 ▸(*e*) and 4 ▸(*f*), with α and β parameters falling in the region identified in the α/β plot.

### 5.3. Blurred cases   

Blurring, in general, should not result in suboptimal refinement. However, blurring would result in smeared and convoluted maps that would be difficult to interpret. Moreover, FFT-based refinement programs would become slow and derivative calculations would be inaccurate. If refinement is carried out carefully (it is software dependent) then the only parameter that would be affected is *B*_0_.

In general, the minimal *B* value is the safest level that can be used for map sharpening and/or refinement. However, it is not easy to derive the minimal *B* value before atomic refinement. Such sharpened maps (using minimal *B* as a sharpening parameter) would show more detail without causing negative *B* values.

**Figure 5:**
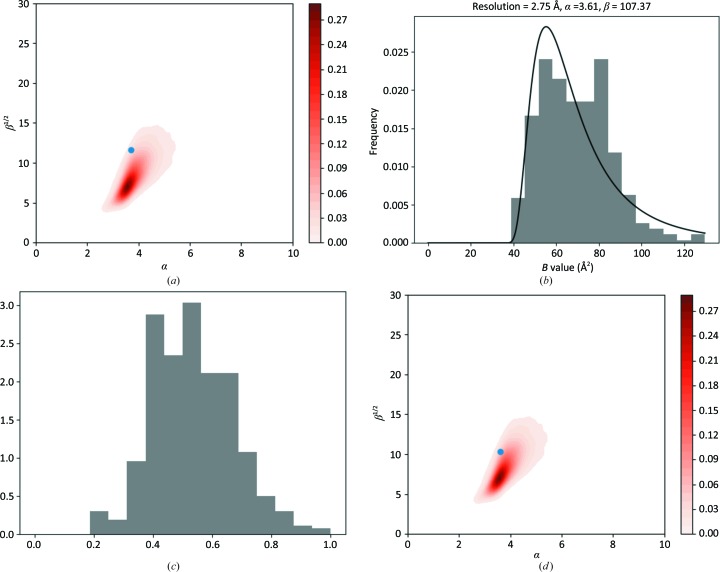
An over-blurred case: PDB entry 5g4t. (*a*) Parameters of the SIGD for PDB entry 5g4t shown on an α/β plot before slight sharpened refinement. (*b*) The *B*-value distribution. (*c*) The peak-height distribution. (*d*) The SIGD parameters on an α/β plot after re-refinement.

An example of an over-blurred case is PDB entry 5g4t (Fig. 5 ▸). In such cases, subtracting a *B* value slightly less than the minimum *B* value for refinement can change refinement behaviour.

### 5.4. Outliers   

**Figure 6:**
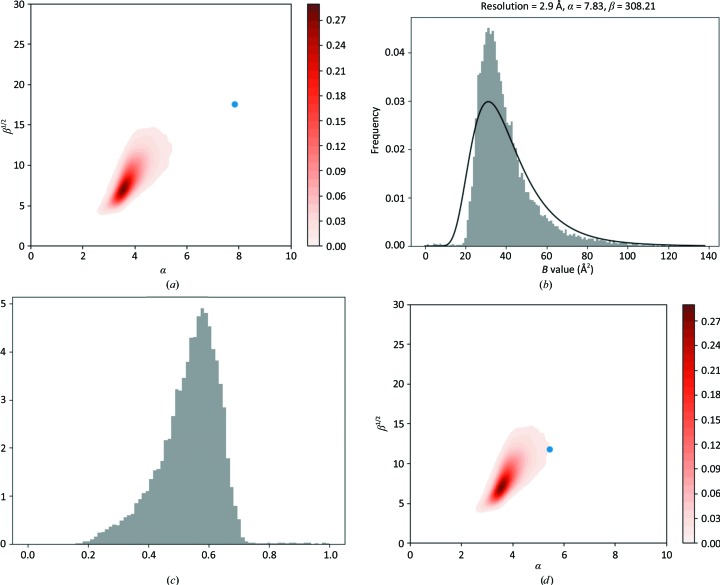

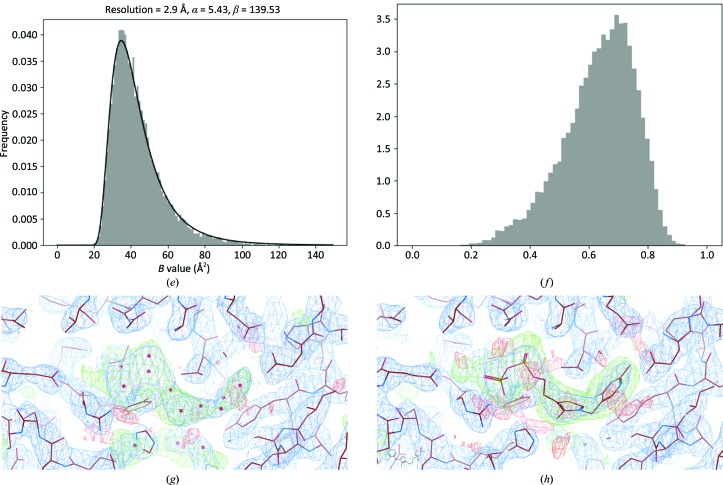
An under-modelled case: PDB entry 2bp7. (*a*) Parameters of the SIGD for PDB entry 2bp7 shown on an α/β plot. (*b*) The SIGD. (*c*) The peak-height distribution. (*d*) The SIGD parameters shown on an α/β plot after model building and refinement. (*e*) The SIGD after model building and refinement. (*f*) The peak-height distribution after model building and refinement. (*g*) The density for a putative ligand before model building and refinement. (*h*) The density for the ligand after model building and refinement. (*g*) and (*h*) were prepared using *CCP*4*mg* (McNicholas *et al.*, 2011 ▸).

There are a number of cases with large α or β values. These are difficult to classify, and there may be various reasons for these outliers. One such case is PDB entry 2bp7: from viewing the parameters of the estimated SIGD on the α/β plot (Fig. 6 ▸ *a*), it is evident that this is a clear outlier with a larger α of 7.83 and a relatively large β of 308.21. The SIGD (Fig. 6 ▸ *b*) and peak-height distributions (Fig. 6 ▸ *c*) show that there are some atoms with small *B* values and therefore relatively large peak heights. Closer analysis revealed that most of the side chains of methionine residues are missing, and that there are some heavier atoms that are interpreted as water molecules. The crystallization conditions indicate that there could be magnesium, sodium, potassium, chlorine and phosphate present as well as molecules of thiamine diphosphate. Inspection of the map showed that these molecules/ions are present in the crystal. After a few rounds of model building and rebuilding, *R* and *R*_free_ decreased from 0.197 and 0.245 to 0.160 and 0.225, respectively (see supporting information). The SIGD parameters estimated using *B* values from the rebuilt structure are close to the ‘acceptable’ region of the α/β plot (Fig. 6 ▸ *d*). The histogram of the *B* values, together with the fitted SIGD (Fig. 6 ▸ *e*) and peak-height distribution (Fig. 6 ▸ *f*), show that there are still some atoms with small *B* values as well as some with large *B* values that need to be removed. Figs. 6 ▸(*g*) and 6 ▸(*h*) show the region of the density before and after rebuilding in the region of the thiamine diphosphate. This example demonstrates how the *B*-value distribution, together with the fitted SIGD parameters, can help to identify some of the shortcomings of atomic models. In particular, very low *B* values can indicate that at least some of the atomic identities are wrong.

### 5.5. Multimodal *B*-value distributions   

There are a number of PDB entries for which the distribution of *B* values exhibits multimodality. There can be various reasons for such behaviour. Two such reasons are the following.

(i) It is an intrinsic property of the molecules within their environment (crystal or multi-domain/multi-subunit structures in cryo-EM), where different components have different numbers of neighbours to interact with. In such cases, different subunits/domains may have different levels of mobility, and this will be reflected in the *B*-value distribution. It can be expected that each individual structural unit will exhibit SIGD behaviour with different parameters. However, the refinement of atomic positions and consequently *B* values in such cases becomes unstable, resulting in inaccurate models. This means that the distribution of *B* values for chains that have higher *B* values may violate the SIGD hypothesis.
(ii) Another common case that exhibits the symptom of multimodal *B*-value distributions is when some parts of the model (loops, ligands or even domains) may have been placed incorrectly. Essentially, such behaviour indicates that there is very weak or no evidence to support the presence of these parts of the structures, and as such they should be considered with extreme care.

**Figure 7:**
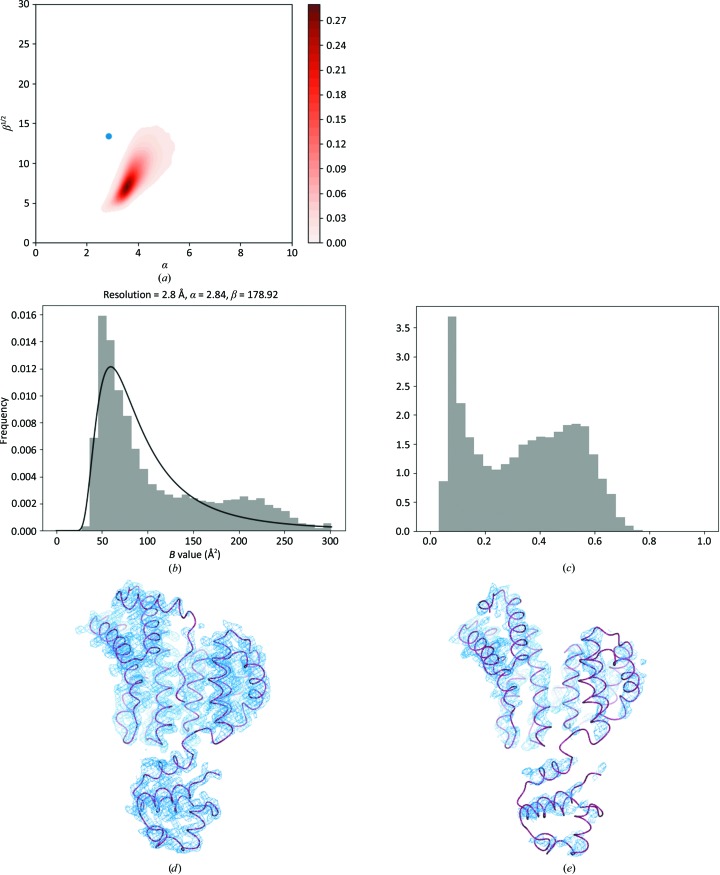
A multimodal *B*-value distribution – chains with different contact numbers: PDB entry 2grm. (*a*) Parameters of the SIGD for PDB entry 2grm shown on an α/β plot. (*b*) The SIGD and *B*-value distribution. (*c*) The peak-height distribution. (*d*, *e*) The density for chains *A* and *C* contoured at σ level 1. *CCP*4*mg* (McNicholas *et al.*, 2011 ▸) was used to produce (*d*) and (*e*).

**Figure 8:**
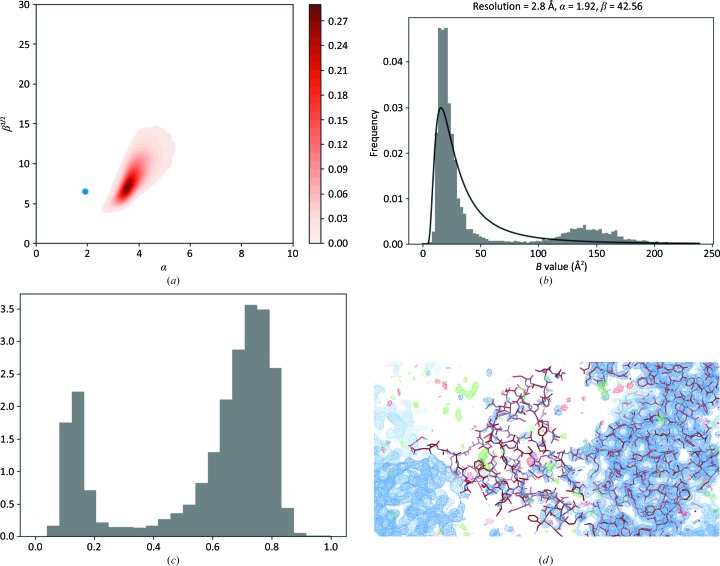
A multimodal *B*-value distribution – disordered region: PDB entry 4l39. (*a*) Parameters of the SIGD for PDB entry 4l39 shown on an α/β plot. (*b*) The SIGD and *B*-value distribution. (*c*) The peak-height distribution. (*d*) The density map, highlighting the various levels of density for the main part of the model and a small domain. The density was contoured at σ level 1. *Coot* (Emsley *et al.*, 2010 ▸) was used to produce (*d*).

Here, we analyse two cases: PDB entry 2grm, where there are two sets of subunits with very different *B* values (Fig. 7 ▸), and PDB entry 4l39, where the density for part of the molecule is very weak (Fig. 8 ▸).

For PDB entry 2grm (Fig. 7 ▸), the *B*-value and peak-height distributions clearly exhibit multimodal distributions (Figs. 7 ▸ *b* and 7 ▸ *c*). The parameters of the SIGD for this case are slightly outside the α/β plot (Fig. 7 ▸ *a*). Analysis of the *B* values shows that chains *A* and *B* have low *B* values and chain *C* has higher *B* values. The density corresponding to chains *A* and *B* is also better than that for chain *C* (Figs. 7 ▸ *d* and 7 ▸ *e*). In this case, it seems that the occurrence of different *B* values for different chains is owing to the fact that they have a different number of contacts to stabilize them within the crystal (Table 3 ▸). Chain *C* interacts only with itself within the asymmetric unit, and makes only 15 interactions with chain *B*. In contrast, chains *A* and *B* interact with each other within and between asymmetric units. Presumably, this is the reason why the mobility of chain *C* is higher than those of the others, resulting in higher overall *B* values. The peak-height distribution (Fig. 7 ▸ *c*) also shows that there is at least one atom that is substantially heavier than the other atoms. Analysis of the density revealed that this atom is on a twofold axis. The PDB header shows that lithium chloride was used for crystallization. If this atom is heavier than the other atoms then it is likely to be a Cl atom, although the identification of Cl atoms is usually very difficult, especially at this resolution (2.8 Å).

In the case of PDB entry 4l39 (Fig. 8 ▸), the higher *B* values, and therefore the more smeared density, are the result of a disordered domain. Inspection of the density (Fig. 8 ▸ *d*) clearly indicates that there might be a different interpretation of this domain and that it might perhaps be better if such loops were left out of the atomic model altogether.

## 6. Conclusions and future perspectives   

We have demonstrated that there is a need to model as well as to validate atomic ADPs. It is demonstrated that for many macromolecular structures the SIGD can be used to model the distribution of ADPs. Even if the *B*-value distribution over the whole structure does not obey the SIGD, the individual chains/domains will obey this distribution. When the distributions of *B* values for different chains/domains are different there can be at least two reasons: (i) different domains/subunits have different contacts depending on the environment and (ii) there are disordered and/or mismodelled regions that have naturally higher *B* values, reflecting errors in the positional parameters. Such multimodality affects the density and therefore the interpretability of the maps. Future work will include the refinement of multimodality parameters (the number of classes and parameters of the SIGD for each class) using such techniques as the expectation-maximization algorithm.

We also show that by modelling the *B*-value distribution using the SIGD and comparing the parameters with those derived from all PDB entries, one can identify the degree of sharpening/blurring before refinement. Whilst both sharpening and blurring are valid procedures that can help in the interpretation of density maps, applying these procedures prior to or during refinement can result in suboptimal atomic models. Too much sharpening results in series-termination and noise-amplification effects that reduce the interpretability of maps, and there might be additional density owing to series-termination effects that can be erroneously modelled as water molecules. In general, it is recommended that during map interpretation, even if sharpening is used, the local contacts must be analysed in order to ensure that the modelled atoms/molecules make chemical sense. During refinement, a reasonable level of sharpening must be used, otherwise refinement can become very slow and convergence may not be reached, or refinement can result in negative *B* values that consequently may affect the density map as well as the refinement procedure.

Since the signal-to-noise ratio in the maps is related to the overall mobility, and therefore the atomic *B* values of the molecules, it is not surprising that when the distribution of the *B* values for a given subunit/domain is shifted to the right (increased) then the corresponding density becomes weaker and the resolvability of peaks is reduced. Estimation of the SIGD parameters can also provide an estimate of the minimal safe *B* value for sharpening. When the data are over-sharpened this affects the distribution of *B* values, moving them to the left (reducing them). In this case, the refinement procedure often becomes stuck, as *B* values cannot become negative. Correcting for over-sharpening, *i.e.* artificially adding a *B* value to the data before refinement, seems to improve the behaviour of refinement, leading to a more reliable atomic model with associated ADPs.

In this work, we also analyse the resolution-dependent peak-height distribution and show that the effect of the *B*-value distribution is dramatically different at different resolutions. In principle, the *B*-value distribution, together with its effect on the peak-height distribution, can be used as a proxy for the resolution/information content of the data: at higher resolution it can be expected that the *B*-value distribution will be narrow and at low resolution it should be wider.

In future, we plan to apply a theoretical *B*-value distribution for the refinement of atomic models at lower resolutions where the data are not sufficient to accurately estimate individual atomic *B* values.

Future work will also consider the potential of using the IGD for map sharpening. The usual map-sharpening technique assumes that there is a single *B* value for all atoms. If we use the distribution of *B* values then we should be able to design better sharpening normalization procedures and therefore better map-sharpening procedures. A similar approach was used by Blessing *et al.* (1996 ▸), under the assumption that the *B*-value distribution follows a normal distribution. We also intend to include local *B*-value analysis together with the effect on peak heights to identify wrongly modelled atoms or regions of the structure.

## Supplementary Material

Refined and rebuilt atomic model for 2bp7.. DOI: 10.1107/S2059798319004807/qh5059sup1.txt

## Figures and Tables

**Table 1:** PDB entries rejected from analysis The main reasons for refinement failures were (i) new ligands not present in the CCP4 monomer library, (ii) amplitudes of structure factors not present in the PDB entry (those with only intensities deposited were excluded from our analysis) and (iii) no experimental data were available. In the final stage, if the highest resolution of the data was higher than 1.5 Å or lower than 3 Å then the entry was removed from further analysis.

No. of remaining entries	Rejected	Reason for rejection
89862	1749	Zero *B* value
88113	1914	Viruses
86299	229	Zero occupancy
86070	261	Space-group incompatibility
85009	19965	Failed refinement
65844	744	High *R* factor
65100	18148	Outside resolution 1.5–3 Å
46952	—	—

**Table 2:** Summary of the PDB entries used as examples *R* and *R*_free_ before, *R* factors before refinement; *R* and *R*_free_ after, *R* factors after refinement.

PDB code	Case	Resolution (Å)	*R* before	*R* after	*R*_free_ before	*R*_free_ after	α	β	*B*_0_ (Å^2^)
1a4i †	Shifted IG distribution	1.50	0.199	0.178	0.235	0.210	3.68	37.45	6.22
1fce	Shifted IG distribution	2.00	0.165	0.129	0.210	0.164	3.60	32.00	6.27
3ad4	Over-sharpening	2.20	0.169	0.176	0.256	0.252	2.54	13.52	−2.20
5g4t	Blur	2.75	0.194	0.219	0.234	0.262	3.71	134.88	66.91
2bp7	Outlier	2.90	0.227	0.193	0.254	0.245	7.83	308.21	−3.58
2grm	Different contact number (bimodality)	2.80	0.256	0.219	0.287	0.274	2.84	178.92	13.00
4l39	Disordered region (bimodality)	2.81	0.198	0.205	0.267	0.264	1.92	42.55	1.47

† In this case only the results of the restrained refinement are given.

**Table 3:** Interactions between chains for PDB entry 2grm The number of interactions (the number of atom pairs with a distance less than 3.6 Å) between chains within the asymmetric unit and via crystallographic symmetry. The first number within each cell is the number of interactions within the asymmetric unit and the second number is that via crystallographic symmetry. The number of intra-chain interactions within the asymmetric unit is set to zero.

Chain	*A*	*B*	*C*
*A*	0, 148	15, 31	0, 0
*B*	15, 31	0, 140	15, 0
*C*	0, 0	15, 0	0, 154

## Footnotes

1 For actual peak analysis one needs to consider the form factors, occupancies, resolution and *B* values of the atoms. However, in this work, our aim is to analyse the relative effects of *B* values and resolution; effectively we normalize the peak heights by the atom types and occupancies, *i.e.* divide the actual peak heights by the occupancies and a factor related to the form factors.

2 At the time of the analysis the program *REFMAC*5 would not use intensities automatically. Since the number of cases in which amplitudes were available was deemed to be sufficient for our analysis, the cases in which only intensities were available were rejected.

## Appendix A Derivatives and expected Fisher information matrix for estimation of the parameters for the inverse-gamma distribution

If the distribution of observed data, *e.g.* *B* values, is a shifted inverse-gamma distribution then, for a given sample of size *N*, the likelihood function will have the form

where the *B_i_* are the *B* values of atoms and α, β and *B*_0_ are the parameters of the SIGD. Taking the logarithm of this expression and multiplying by −1 results in the negative log-likelihood function,

We want to use Fisher’s scoring method with the expected Fisher information matrix to find the minimum of the negative log-likelihood function (Stuart *et al.*, 1999 ▸; Steiner *et al.*, 2003 ▸). The first derivatives of this function with respect to the parameters of the distribution have the form

In the above expression ψ(α) = Γ′(α)/Γ(α) is the psi function, which is the derivative of the log of the gamma function. The first derivative of the psi function is called the di-gamma function, with further derivatives being called poly-gamma functions (Abramowitz & Stegun, 1964 ▸). In principle, these derivatives should be sufficient to use first-order methods such as the steepest-descent and conjugate-gradient methods for parameter estimation. However, to use the second-order methods we need to calculate the second derivatives. We prefer using the Fisher scoring method with the expected Fisher information matrix, the main reason being that the Fisher information matrix is non-negative. As a result, shifts based on the Fisher information matrix are always directed towards the minima of the negative log-likelihood function.

Let us use the notation **I** for Fisher’s expected information matrix, with elements *I_ij_* = *E*(∂^2^ *l*/∂*p_i_*∂*p_j_*) (Stuart *et al.*, 1999 ▸) with (*p*_1_, *p*_2_, *p*_3_) denoting the (α, β, *B*_0_) parameters of the IG. It can be shown that elements of the information matrix can be calculated using

For the derivation of these formulae, we used expressions for the moments of the IGD: *E*(*x^n^*) = β^*n*^Γ(α + *n*)/Γ(α) valid for *n* < α, noting that this formula is valid for all negative values of *n*.
